# Comparison of nine trauma scoring systems in prediction of inhospital outcomes of pediatric trauma patients: a multicenter study

**DOI:** 10.1038/s41598-024-58373-4

**Published:** 2024-04-01

**Authors:** Armin Khavandegar, Payman Salamati, Mohammadreza Zafarghandi, Vafa Rahimi-Movaghar, Mahdi Sharif-Alhoseini, Esmaeil Fakharian, Seyed Houssein Saeed-Banadaky, Vahid Hoseinpour, Farideh Sadeghian, Mehdi Nasr Isfahani, Vahid Rahmanian, Amir Ghadiphasha, Sobhan Pourmasjedi, Seyed Mohammad Piri, Sara Mirzamohamadi, Mahgol Sadat Hassan Zadeh Tabatabaei, Khatereh Naghdi, Vali Baigi

**Affiliations:** 1 Sina Trauma and Surgery Research Center, Tehran University of Medical Sciences, Tehran, Iran; 2 Trauma Research Center, Kashan University of Medical Sciences, Kashan, Iran; 3 Trauma Research Center, Rahnemoon Hospital, School of Medicine, Shahid Sadoughi University of Medical Sciences, Yazd, Iran; 4 Department of Emergency Medicine, School of Medicine, Urmia University of Medical Sciences, Urmia, Iran; 5 Center for Health Related Social and Behavioral Sciences Research, Shahroud University of Medical Sciences, Shahroud, Iran; 6 Department of Emergency Medicine, Faculty of Medicine, Isfahan University of Medical Sciences, Isfahan, Iran; 7 Trauma Data Registration Center, Isfahan University of Medical Sciences, Isfahan, Iran; 8 Research Center for Social Determinants of Health, Jahrom University of Medical Sciences, Jahrom, Iran; 9 Shahid Modarres Hospital, Saveh University of Medical Sciences, Saveh, Iran; 10 Department of Epidemiology and Biostatistics, School of Public Health, Tehran University of Medical Science, Tehran, Iran

**Keywords:** Glasgow coma scale (GCS), Pediatric trauma score, Injury severity score, Trauma scoring system, Trauma and injury severity score, Survival prediction model, Children, Trauma, Paediatric research, Epidemiology

## Abstract

Hereby, we aimed to comprehensively compare different scoring systems for pediatric trauma and their ability to predict in-hospital mortality and intensive care unit (ICU) admission. The current registry-based multicenter study encompassed a comprehensive dataset of 6709 pediatric trauma patients aged ≤ 18 years from July 2016 to September 2023. To ascertain the predictive efficacy of the scoring systems, the area under the receiver operating characteristic curve (AUC) was calculated**.** A total of 720 individuals (10.7%) required admission to the ICU. The mortality rate was 1.1% (n = 72). The most predictive scoring system for in-hospital mortality was the adjusted trauma and injury severity score (aTRISS) (AUC = 0.982), followed by trauma and injury severity score (TRISS) (AUC = 0.980), new trauma and injury severity score (NTRISS) (AUC = 0.972), Glasgow coma scale (GCS) (AUC = 0.9546), revised trauma score (RTS) (AUC = 0.944), pre-hospital index (PHI) (AUC = 0.936), injury severity score (ISS) (AUC = 0.901), new injury severity score (NISS) (AUC = 0.900), and abbreviated injury scale (AIS) (AUC = 0.734). Given the predictive performance of the scoring systems for ICU admission, NTRISS had the highest predictive performance (AUC = 0.837), followed by aTRISS (AUC = 0.836), TRISS (AUC = 0.823), ISS (AUC = 0.807), NISS (AUC = 0.805), GCS (AUC = 0.735), RTS (AUC = 0.698), PHI (AUC = 0.662), and AIS (AUC = 0.651). In the present study, we concluded the superiority of the TRISS and its two derived counterparts, aTRISS and NTRISS, compared to other scoring systems, to efficiently discerning individuals who possess a heightened susceptibility to unfavorable consequences. The significance of these findings underscores the necessity of incorporating these metrics into the realm of clinical practice.

## Introduction

Trauma is the primary cause of mortality in children, and extensive research has been conducted to identify effective strategies for reducing the resulting morbidity and mortality rates^1,2^. Accurately measuring the severity of injuries is crucial for assessing the quality of care provided to children with trauma and for conducting research on their outcomes^3^. Despite the guidelines put forth by the American College of Surgeons (ACS) Committee on Trauma (COT), the triage of children still exhibits considerable inconsistency. At present, there is a lack of consensus on a universally accepted pediatric triage scoring system^4^. To address this issue and to facilitate the prompt assessment and effective allocation of resources for pediatric trauma patients, scoring systems have been employed to ensure efficient and accurate decision-making in trauma patients^4^.

Scoring systems have been classically classified as anatomical, physiological, or combined scoring systems^5,6^. The AIS, ISS, and NISS are anatomical scoring systems that employ anatomical variables, including the location and severity of injury^7^. The GCS, RTS, and PHI are among the physiological scoring systems used and can be calculated through values retrieved from physical examination data^7,8^. Finally, the TRISS, NTRISS, and aTRISS are combined scoring systems that utilize both anatomical and physiological features of trauma^9^. The GCS is primarily utilized to evaluate the level of consciousness impairment in patients, achieved through the assessment of ocular, motor, and verbal responses^10^. The abbreviated injury AIS has also been employed in pediatric trauma by appointing a value of one to six to the injury^10^. The two main derivates of the AIS, ISS and NISS, were also utilized to predict pediatric outcomes following trauma based on the three injuries with the highest scores^10,11^. Similarly, the TRISS showed acceptable predictive performance for pediatric trauma outcomes^11^. RTS and PHI play significant roles in improving pediatric trauma triage^4,8,12^.

The AIS was first developed in 1969^13^. After that, in 1974, simultaneously, the GCS was announced by Teasdale and Jennett in Scotland^14^, and the ISS was established by Baker to address the cumulative effect of injury, which was missed in conventional AIS^15^. Seven years after that, in 1981, the first version of the TRISS was introduced, claiming that the combined use of physiological and anatomical indices in addition to the age range is a very powerful tool for survival prediction^16^. In 1986, the PHI was developed primarily based on four items as a triage-based trauma scoring system^17^. In 1989, a revision of the trauma score named the RTS, was introduced^18^. In 1997, Osler et al. provided a new version of the ISS called the NISS^19^. Eventually, in 2018, a novel modified version of the TRISS, called the NTRISS, was introduced by Domingues et al.^9^.

During the initial assessment of a trauma patient, it is crucial to predict various factors, including the need for intensive care and the potential for morbidity and mortality. The objective of this study was to ascertain the prognostic significance of commonly utilized trauma scores, specifically in pediatric patients. We used the National Trauma Registry of Iran (NTRI) to perform a comprehensive comparison among the AIS, ISS, NISS, RTS, PHI, TRISS, and two of its variants (aTRISS and NTRISS) scoring systems for pediatric trauma and evaluated their ability to predict in-hospital mortality and ICU admission.

## Methods and materials

### Study design and population

This retrospective registry-based study was conducted at eight renowned trauma care hospitals in Iran. The current study encompassed a comprehensive dataset of 6709 pediatric trauma patients aged ≤ 18 years from July 2016 to September 2023. The traumatic events referred to in this context are those specifically outlined in Chapter XX of the International Statistical Classification of Diseases and Health-Related Problems (ICD-10). All the patients in the study were evaluated in an emergency department at one of the hospitals. Patients who were discharged home from the emergency department were excluded from this study. Individuals who were transferred out were excluded from this study due to the potential lack of comprehensive evaluations before their transfer, and their subsequent survival status remains uncertain. Mode of transport was defined as the way the victim was transported from the accident site to the hospital and was categorized as a private vehicle, Emergency Medical services (EMS), and others. In this study, multiple trauma was defined as injury to more than one site, regardless of their severity. The main cause of trauma was divided into transportation accidents, falls, and others. The primary outcomes of this study were ICU admission and in-hospital mortality, defined as occurring either in the emergency department (ED) or during the patient's hospital stay.

### Scoring system calculations

The details of the scoring system calculations are described in Supplementary Table 1.

### Determination of cutoff points for each scoring system

After conducting a receiver operating characteristic (ROC) curve analysis for each scoring system and thoroughly reviewing the relevant literature, the following cutoff points were selected: AIS ≥ 3 compared to AIS < 3, RTS < 7 (vs. ≥ 7), GCS ≤ 8 (vs. > 8), PHI ≥ 3 (vs. < 3), TRISS, aTRISS, and NTRISS < 0.9 (vs. ≥ 0.9). Due to the multitude of different cutoff points found in the literature, we utilized two cutoff points for the ISS and NISS (≥ 16 vs < 16 and ≥ 25 vs. <  25).

### Statistical analysis

Numbers and percentages were used to describe nominal and categorical variables, respectively. Continuous variables were described as mean ± standard deviation (SD) in the case of the normal distribution; otherwise, they were presented as median and interquartile range (IQR). Univariate and multiple logistic regression models were employed to evaluate the factors influencing hospital mortality and ICU admission. To ascertain the predictive efficacy of the scoring systems, the AUC was calculated. To examine the association between the identified cutoff points and the two main outcomes, crude binary logistic regression was initially conducted. Subsequently, adjusted binary logistic regression was performed after controlling for confounding factors encompassing age, sex, and mechanism of trauma. The equality of the AUC was tested using Stata's roccomp command. It provides summary statistics such as the AUC, standard errors, confidence intervals, and p-values for the DeLong test. The DeLong test, provided by roccomp, assesses the null hypothesis that the AUCs of the two models are equal. Data analysis was conducted using Stata software version 14.0 (Stata Corp., College Station, TX, USA; Available on https://www.stata.com/stata14/).

## Results

### Baseline and clinical characteristics of patients

Among the 6709 pediatric trauma patients, 5309 (79.3%) were male. The mean (SD) age of the included patients was 11.85 (5.88). The main cause of trauma was transportation accidents, accounting for 3401 patients (50.7%), followed by falls (1623 patients, 24.2%). Blunt trauma was the most prevalent type of injury, affecting 5888 patients (87.8%). In terms of transportation to the hospital, 3383 patients (50.4%) were transported via EMS, while 3194 patients (47.6%) relied on private vehicles.

A total of 2527 patients, accounting for 37.7% of the total patients, were identified as having multiple traumatic events. Among the reported cases, injuries to the extremities were the most common, affecting 2993 individuals or 44.6%. This was followed by isolated injuries to the head, face, and neck, which were reported in 768 patients (11.4%).

Among the total patients, 720 (10.7%) required admission to the ICU. A total of 72 patients (1.1%) died during hospitalization. The mortality rate in the ICU was 7.2% (n = 52). The median length of stay (LOS) for all patients was 2 days, with an IQR of 1 to 4 days. However, for those admitted to the ICU, the median LOS was 4 days, with an IQR of 2 to 7 days.

### Scoring systems

The median (IQR) of all scoring systems is presented in Table 1. As demonstrated in Table 1, all nine measured scores were significantly different among in-hospital deceased and nondeceased patients and between ICU-admitted and non-ICU-admitted pediatric trauma patients (p < 0.001).

**Table 1:** Comparison of median (IQR) values for each scoring system based on two main outcomes.

Scoring systems	In-hospital mortality			ICU admission		
	Yes	No	p value^†^	Yes	No	p value^†^
AIS	3.00 (2.00)	2.00 (1.00)	< 0.001	2.00 (1.00)	2.00 (1.00)	< 0.001
ISS	16.00 (18.00)	4.00 (3.00)	< 0.001	9.00 (9.00)	4.00 (3.00)	< 0.001
NISS	18.00 (24.00)	4.00 (7.00)	< 0.001	12.00 (13.00)	4.00 (6.00)	< 0.001
RTS	4.30 (2.40)	7.84 (0)	< 0.001	7.84 (1.87)	7.84 (0)	< 0.001
GCS	4.00 (4.00)	15.00 (0)	< 0.001	14.00 (7.00)	15.00 (0)	< 0.001
TRISS	0.866 (0.403)	0.996 (0.003)	< 0.001	0.991 (0.022)	0.996 (0.002)	< 0.001
NTRISS	0.826 (0.420)	0.995 (0)	< 0.001	0.988 (0.040)	0.995 (0)	< 0.001
aTRISS	0.789 (0.570)	0.994 (0.004)	< 0.001	0.987 (0.037)	0.994 (0.002)	< 0.001
PHI	9.00 (5.00)	3.00 (1.00)	< 0.001	4.00 (5.00)	3.00 (1.00)	< 0.001

† Mann‒Whitney test was utilized.

*IQR* interquartile range, *ICU* intensive care unit, *AIS* abbreviated injury scale, *ISS* injury severity score, *NISS* new injury severity score, *RTS* revised trauma score, *GCS* Glasgow coma scale, *TRISS* trauma and injury severity score, *NTRISS* new trauma and injury severity score, *aTRISS* adjusted trauma and injury severity score, *PHI* prehospital index.

### Predictive performance of the scoring systems for pediatric in-hospital mortality

**Figure 1:**
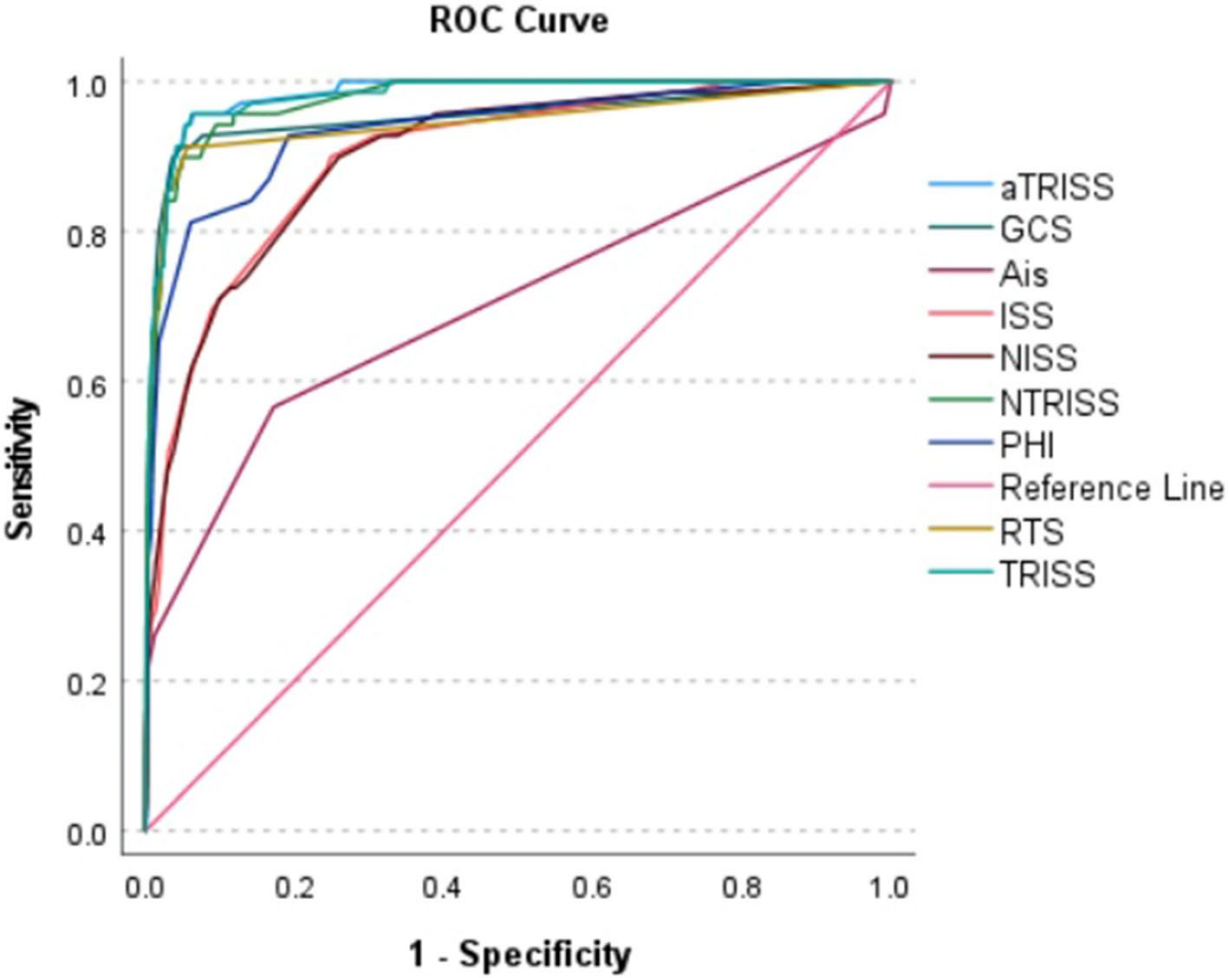
ROC curves for all nine scoring systems in the prediction of in-hospital mortality.

According to the findings presented in Table 2, the system that demonstrated the highest predictive performance for in-hospital mortality was aTRISS, with an AUC value of 0.982. The next highest predictive performances belong to TRISS (AUC = 0.979), NTRISS (AUC = 0.972), GCS (AUC = 0.955), RTS (AUC = 0.944), PHI (AUC = 0.936), ISS (AUC = 0.901), NISS (AUC = 0.900), and AIS (AUC = 0.735). Figure 1 shows the ROC curves for all nine scoring systems for the prediction of in-hospital mortality.

**Table 2:** Performance of scoring systems in prediction of in-hospital mortality and ICU admission.

Scoring systems	N	AUC (95% CI)	
		In-hospital mortality	ICU admission
AIS	6642	0.735 (0.664–0.805)	0.651 (0.629–0.672)
ISS	6707	0.901 (0.866–0.935)	0.807 (0.789–0.825)
NISS	6709	0.900 (0.865–0.936)	0.805 (0.787–0.824)
RTS	6589	0.944 (0.909–0.980)	0.698 (0.679–0.717)
GCS	6669	0.955 (0.923–0.986)	0.738 (0.719–0.757)
TRISS	6589	0.980 (0.968–0.992)	0.823 (0.804–0.842)
NTRISS	6663	0.972 (0.959–0.986)	0.837 (0.820–0.854)
aTRISS	6589	0.982 (0.972–0.991)	0.836 (0.818–0.854)
PHI	6528	0.936 (0.902–0.969)	0.662 (0.638–0.686)

*AIS* abbreviated injury scale, *ISS* injury severity score, *NISS* new injury severity score, *RTS* revised trauma score, *GCS* Glasgow coma scale, *TRISS* trauma and injury severity score, *NTRISS* new trauma and injury severity score, *aTRISS* adjusted trauma and injury severity score, *PHI* prehospital index, *CI* Confidence Interval, *AUC* Area Under Receiver Operating Characteristic curve.

### Predictive performance of the scoring systems for pediatric ICU admission

**Figure 2:**
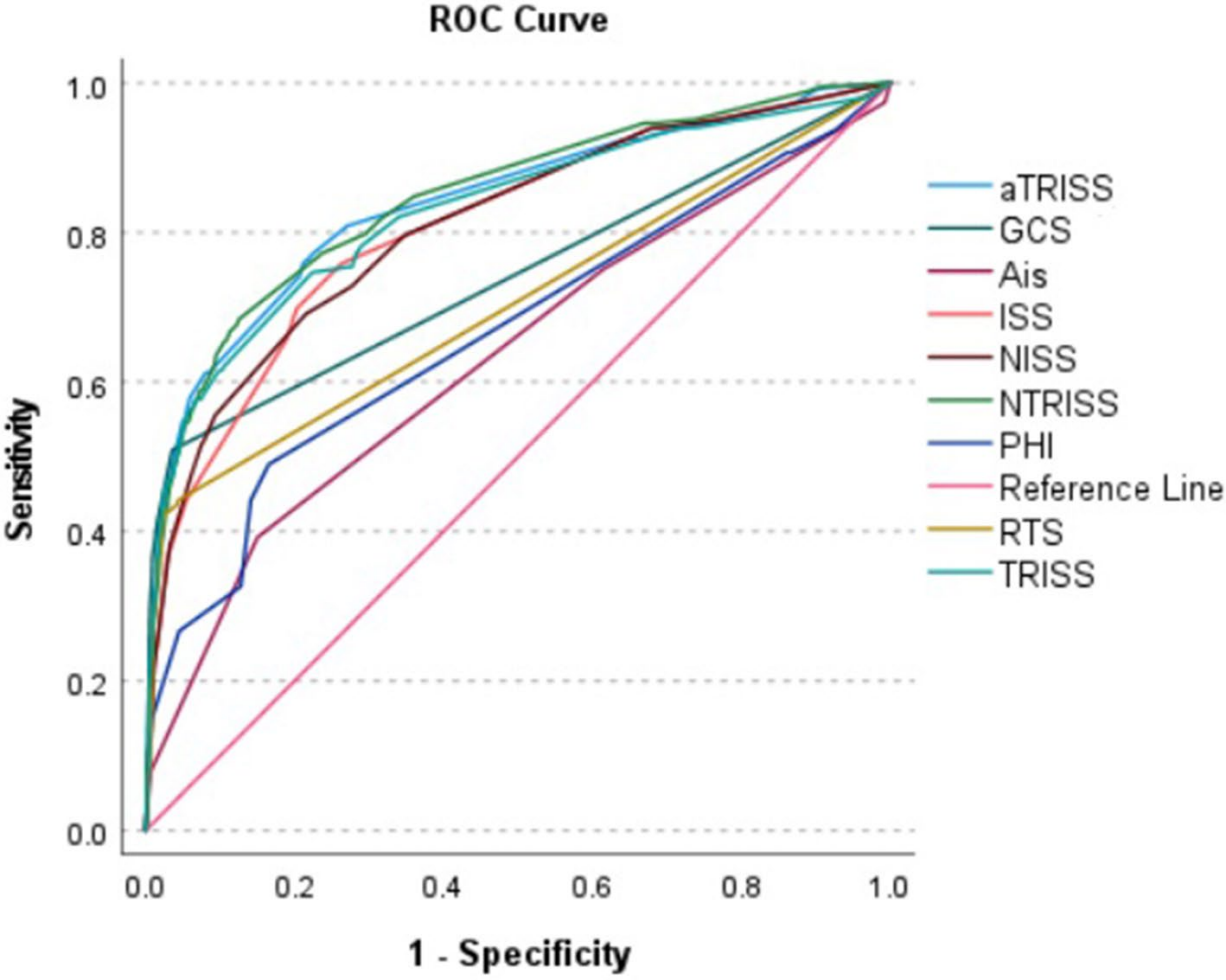
ROC curves for all nine scoring systems in the prediction of ICU admission.

As unveiled in Table 2, the NTRISS had the highest predictive performance for ICU admission, with an AUC of 0.844. Closely followed by aTRISS, with an AUC of 0.836, TRISS at 0.823, ISS at 0.807, NISS at 0.805, GCS at 0.735, RTS at 0.698, PHI at 0.662, and AIS at 0.651. Figure 2 shows the ROC curves for all nine scoring systems for the prediction of ICU admission.

### Crude logistic regression of the association between the categorized scoring systems and the two main outcomes

As illustrated in Table 3, the odds of death and ICU admission in patients with AIS ≥ 3 were 7.19 and 3.82 times greater than those in patients with AIS < 3. The odds of death in patients with ISS and NISS ≥ 16 were 7.80 and 7.20 times greater than those in patients with ISS and NISS < 16. The highest odds ratio (OR) of in-hospital mortality were 216.2 in cases with GCS ≤ 8, followed by OR:106.9 in NTRISS < 0.9 and OR: 104.40 in RTS < 7. The highest odds of ICU admission were attributed to patients with aTRISS < 0.9 (OR = 27.4), followed by those with a GCS ≤ 8 (OR = 26.7) and those with a RTS < 7 (OR = 25.3).

**Table 3:** Crude logistic regression of determined cutoffs for each pediatric scoring system for two main outcomes.

Cutoff scores	n	In-hospital mortality			ICU admission		
		OR	95% CI	p value	OR	95% CI	p value
AIS ≥ 3	1187	7.19	4.41–11.74	< 0.001	3.82	3.23–4.51	< 0.001
ISS							
≥ 16	247	7.80	4.46–13.65	< 0.001	17.92	13.45–23.88	< 0.001
≥ 25	68	13.37	6.99–25.59	< 0.001	12.86	7.34–22.52	< 0.001
NISS							
≥ 16	470	7.20	4.02–12.92	< 0.001	15.35	12.43–18.96	< 0.001
≥ 25	123	8.91	4.99–15.90	< 0.001	18.15	11.96–27.54	< 0.001
RTS < 7	442	104.40	40.93–266.34	< 0.001	25.53	20.24–32.19	< 0.001
GCS ≤ 8	226	216.21	111.71–418.46	< 0.001	26.71	21.02–33.94	< 0.001
TRISS < 0.9	74	74.34	38.53–143.43	< 0.001	13.58	7.36–25.07	< 0.001
NTRISS < 0.9	167	106.99	62.86–182.11	< 0.001	8.99	6.68–12.09	< 0.001
aTRISS < 0.9	118	68.87	34.20–138.69	< 0.001	27.34	16.57–45.10	< 0.001
PHI ≥ 4	2761	19.35	6.04–62.04	< 0.001	2.28	1.93–2.70	< 0.001

*ICU* intensive care unit, *AIS* abbreviated injury scale, *ISS* injury severity score, *NISS* new injury severity score, *RTS* revised trauma score, *GCS* Glasgow coma scale, *TRISS* trauma and injury severity score, *NTRISS* new trauma and injury severity score, *aTRISS* adjusted trauma and injury severity score, *PHI* prehospital index, *CI* Confidence Interval, *OR* odds ratio.

### Multiple logistic regression of the association between the categorized scoring systems and the two main outcomes

According to the data presented in Table 4, after controlling for age, sex, and mechanism of trauma, patients with TRISS, aTRISS, or NTRISS values less than 0.9 had significantly increased odds of in-hospital mortality (Adjusted ORs: 219.9, 173.7, and 107.2, respectively). Additionally, these patients also had greater odds of requiring admission to the ICU (aORs: 28.6, 38.6, and 19.6, respectively) (p < 0.001).

**Table 4:** Multiple logistic regression of determined cutoffs for each pediatric scoring system for two main outcomes (after adjustment for age, gender, and mechanism of trauma).

Cutoff scores	n	In-hospital mortality			ICU admission		
		aOR	95% CI	p value	aOR	95% CI	p value
AIS ≥ 3	1187	6.43	3.91–10.55	< 0.001	3.45	2.92–4.09	< 0.001
ISS							
≥ 16	247	26.65	16.32–43.51	< 0.001	20.34	15.27–27.10	< 0.001
≥ 25	68	49.72	27.10–91.21	< 0.001	20.23	11.81–34.66	< 0.001
NISS							
≥ 16	470	20.69	12.50–34.28	< 0.001	15.42	12.48–19.06	< 0.001
≥ 25	123	33.21	19.51–56.52	< 0.001	22.54	14.94–34.02	< 0.001
RTS < 7	442	167.67	71.85–391.27	< 0.001	30.35	24.00–38.37	< 0.001
GCS ≤ 8	226	199.79	102.37–389.93	< 0.001	61.75	42.26–90.23	< 0.001
TRISS < 0.9	74	219.94	121.17–399.24	< 0.001	28.61	16.29–50.26	< 0.001
NTRISS < 0.9	167	107.21	61.86–186.00	< 0.001	19.64	13.91–27.73	< 0.001
aTRISS < 0.9	118	173.72	98.21–307.31	< 0.001	38.58	23.87–62.35	< 0.001
PHI ≥ 4	2761	43.82	13.66–140.55	< 0.001	3.46	2.91–4.12	< 0.001

*ICU* intensive care unit, *AIS* abbreviated injury scale, *ISS* injury severity score, *NISS* new injury severity score, *RTS* revised trauma score, *GCS* Glasgow coma scale, *TRISS* trauma and injury severity score, *NTRISS* new trauma and injury severity score, *aTRISS* adjusted trauma and injury severity score, *PHI* prehospital index, *CI* Confidence Interval, *OR* odds ratio, aOR adjusted OR.

Furthermore, patients with a GCS ≤ 8 had significantly greater odds of in-hospital mortality (aOR: 199.8) and ICU admission (aOR: 61.7) (p < 0.001). Moreover, in pediatric trauma patients, those with ISS and NISS values equal to or greater than 25 had 49.7 and 33.2 times greater odds of death, respectively, than did their counterparts with ISS and NISS values less than 25. Similarly, these patients also had 20.2 and 22.5 times greater odds of requiring ICU admission, respectively.

### Comparison of paired samples area difference under the ROC among all scoring systems

As displayed in Table 5, it is worth noting that the AIS exhibited considerably poorer predictive performance than did the other eight scoring systems, with p values indicating statistical significance (p < 0.001). Compared with the NTRISS, TRISS, and aTRISS, the RTS exhibited significantly poorer predictive performance (p < 0.05). Eventually, the NTISS had a poorer predictive performance compared to aTRISS (p < 0.05).

**Table 5:** Comparison of paired samples area difference under the ROC curves among all scoring systems.

	Outcomes	aTRISS (a^†^)	NTRISS (b)	TRISS (c)	GCS (d)	RTS (e)	PHI (f)	ISS (g)	NISS (h)	AIS (i)
aTRISS (a)	Death	1	0.020* (a)	0.119	0.042* (a)	0.012* (a)	0.002* (a)	< 0.001* (a)	< 0.001* (a)	< 0.001* (a)
	ICU admission		< 0.001* (b)	< 0.001* (a)	< 0.001* (a)	< 0.001* (a)	< 0.001* (a)	< 0.001* (a)	< 0.001* (a)	< 0.001* (a)
NTRISS (b)	Death	0.020* (a)	1	0.063	0.140	0.047* (b)	0.010* (b)	< 0.001* (b)	< 0.001* (b)	< 0.001* (b)
	ICU admission	< 0.001* (b)		0.002* (b)	< 0.001* (b)	< 0.001* (b)	< 0.001* (b)	< 0.001* (b)	< 0.001* (b)	< 0.001* (b)
TRISS (c)	Death	0.119	0.063	1	0.052	0.014* (c)	0.002* (c)	< 0.001* (c)	< 0.001* (c)	< 0.001* (c)
	ICU admission	< 0.001* (a)	0.002* (b)		< 0.001* (c)	< 0.001* (c)	< 0.001* (c)	0.006* (c)	0.011* (c)	< 0.001* (c)
GCS (d)	Death	0.042* (a)	0.140	0.052	1	0.239	0.032* (d)	0.038* (d)	0.038* (d)	< 0.001* (d)
	ICU admission	< 0.001* (a)	< 0.001* (b)	< 0.001* (c)		< 0.001* (d)	< 0.001* (d)	< 0.001* (g)	< 0.001* (h)	< 0.001* (d)
RTS (e)	Death	0.012* (a)	0.047* (b)	0.014* (c)	0.239	1	0.414	0.088	0.085	< 0.001* (e)
	ICU admission	< 0.001* (a)	< 0.001* (b)	< 0.001* (c)	< 0.001* (d)		< 0.001* (e)	< 0.001* (g)	< 0.001* (h)	< 0.001* (e)
PHI (f)	Death	0.002* (a)	0.010* (b)	0.002* (c)	0.032* (d)	0.414	1	0.156	0.164	< 0.001* (f)
	ICU admission	< 0.001* (a)	< 0.001* (b)	< 0.001* (c)	< 0.001* (d)	< 0.001* (e)		< 0.001* (g)	< 0.001* (h)	0.044 (f)
ISS (g)	Death	< 0.001* (a)	< 0.001* (b)	< 0.001* (c)	0.038* (d)	0.088	0.156	1	0.938	< 0.001* (g)
	ICU admission	< 0.001* (a)	< 0.001* (b)	0.006 (c)	< 0.001* (g)	< 0.001* (g)	< 0.001* (g)		0.817	< 0.001* (g)
NISS (h)	Death	< 0.001* (a)	< 0.001* (b)	< 0.001* (c)	0.038* (d)	0.085	0.164	0.938	1	< 0.001* (h)
	ICU admission	< 0.001* (a)	< 0.001* (b)	0.011* (c)	< 0.001* (h)	< 0.001* (h)	< 0.001* (h)	0.817		< 0.001* (h)
AIS (i)	Death	< 0.001* (a)	< 0.001* (b)	< 0.001* (c)	< 0.001* (d)	< 0.001* (e)	< 0.001* (f)	< 0.001* (g)	< 0.001* (h)	1
	ICU admission	< 0.001* (a)	< 0.001* (b)	< 0.001* (c)	< 0.001* (d)	< 0.001* (e)	0.044 (f)	< 0.001* (g)	< 0.001* (h)	

^†^Lower case alphabets written in each cell shows the superior scoring system in case of statistically significant difference between them.

*Statistically significant.

*ICU* intensive care unit, *AIS* abbreviated injury scale, *ISS* injury severity score, *NISS* new injury severity score, *RTS* revised trauma score, *GCS* Glasgow coma scale, *TRISS* trauma and injury severity score, *NTRISS* new trauma and injury severity score, *aTRISS* adjusted trauma and injury severity score, *PHI* prehospital index.

Besides, as presented in Table 5, the AIS falls short of all its counterpartying scoring systems in terms of predictive performance (p < 0.05). The ISS and NISS both had poorer predictive performance than did the aTRISS and NTRISS (p < 0.001). The TRISS had considerably less predictive performance than did the aTRISS or NTRISS (p < 0.05), and the aTRISS had significantly less predictive performance than the NTRISS (p < 0.05).

### Ethical approval and consent to participate

All ethical and moral issues were considered in this study. Informed consent was obtained from the patients or their next of kin. This study was approved by the ethics committee of Sina Hospital, Tehran University of Medical Sciences (Approval ID:IR.TUMS.SINAHOSPITAL.REC.1399.090). We confirm that all methods were performed in accordance with the relevant guidelines and regulations.

### Consent for publication

Due to the registry-based nature of this study, verbal consent was obtained from the patients of their next of kin, at the time of the interview.

## Discussion

Trauma continues to be the leading cause of death in children, and the progress made in the field of pediatric trauma has closely paralleled advancements in adult trauma care. However, there is considerable disparity among trauma centers in regard to prehospital triage systems^20,21^. In this study, we analyzed various trauma scores of 6709 pediatric trauma patients, namely, AIS, ISS, NISS, RTS, GCS, PHI, TRISS, aTRISS, and NTRISS scores, to establish correlations between these scores and their respective clinical outcomes. The ROC curves demonstrated that the TRISS and its two derivatives, the aTRISS and NTRISS, respectively, possess the inherent capability to accurately distinguish patients who face an elevated likelihood of encountering unfavorable consequences, encompassing in-hospital mortality and ICU admission. These compelling findings underscore the utmost importance of incorporating these measures into clinical practice, thereby elevating the standard of patient care and prognosis. Furthermore, considering anatomical and physiological factors alone for the prediction of pediatric trauma cases may not seem sufficient; however, a combinatorial approach of utilizing both factors simultaneously may increase outcome prediction.

The Abbreviated Injury Scale (AIS) is a scoring system that categorizes injuries based on their severity. A six-point scale ranging from one (minor) to six (maximal) was used to classify injuries according to their relative severity in different body regions. Derived from expert consensus, this system has achieved global recognition^22^. Although intended for all age groups, the AIS does not encompass any specific AIS scores applicable solely to pediatric patients^23^.

The ISS, which was created by Susan Baker in 1974, has been widely employed in the pediatric literature as a measure of injury severity for the past four decades. The ISS (ranging from 1 to 75) was calculated by adding up the squares of the highest AIS severity scores from the three most severely injured body regions. However, if the AIS score is 6, the ISS is automatically set to 75^15,24^. According to a study of 545,015 pediatric blunt trauma patients, the AUC of the ISS for in-hospital mortality prediction was 0.86. The difference between the current study AUC for the ISS (AUC = 0.9006) and the aforementioned study can be explained by the inclusion of penetrating trauma in this study^3^. The conventional ISS assigns equal weight to various body regions. The NISS was developed to address this issue. In addition, the impact of age differences on the severity of disease experienced by patients is neglected in ISS^3^. The TRISS (and its derivatives) were developed to address this.

In 1997. Osler et al. introduced the NISS, which comprises the cumulative value of the squared AIS score pertaining to the three most critical injuries sustained by a patient, irrespective of the affected anatomical region^19^. On the basis of retrospective examination of 6585 adult individuals affected by trauma, Osler et al. revealed that the NISS was better able to predict patient prognosis than the ISS. They used data from two datasets, 3136 patients from Albuquerque Center and 3449 patients from Emanuel Center. The AUC for ISS and NISS was 0.869 and 0.896 for the first center and 0.907 and 0.896 for the second center. They also stated that the difference between these two scoring systems was statistically significant despite overlapping confidence intervals. Our findings implied that there was no significant difference in the prediction of poorer outcomes between the NISS and ISS. Like our findings, in a study by Grisoni et al., which was conducted on 9151 pediatric trauma patients in four different regional trauma centers in the U.S., the disparities in the predictive capabilities of the two scoring systems were deemed inconsequential, and they concluded that in pediatric trauma patients, there are no notable disparities in the predictive capabilities between the ISS and NISS, as reported in studies involving adult trauma patients^25^.

The PHI components are SBP, PR, RR, level of consciousness, and nature of the injury (blunt or penetrating). The concept of the PHI was initially introduced by Koehler et al. and has since gained widespread use in medical triage^17^. The PHI previously exhibited an AUC of 0.926^8^ for the prediction of death following adult trauma.

According to a meta-analysis of 11 relevant studies published in 2019 on the performance of the RTS in predicting in-hospital mortality in a mixed population of adults and children, the overall AUC of the RTS was 0.93^26^, which was similar to our finding (AUC = 0.9444). In another multicenter study of 814 pediatric trauma patients, the AUC of the RTS was 0.949.

In a multicenter study of 45,377 pediatric trauma patients, 2579 were deceased. The AUC for the GCS for the prediction of in-hospital mortality was 0.89^27^. In a study of 104,035 records of pediatric trauma aged 1 to 18 years with 3946 deaths, the AUC of the GCS for mortality prediction was 0.946^28^, which was much closer to that of our study (AUC of GCS for mortality: 0.954)^29^.

The TRISS is extensively employed as the predominant tool for determining the likelihood of patient survival following traumatic injuries^30^. The coefficients of the currently utilized TRISS model were computed using the dataset obtained from the Major Trauma Outcome Study, which was coordinated by the American College of Surgeons Committee on Trauma between 1982 and 1987^31^. Several studies have indicated that the TRISS model may not be appropriate for assessing survival outcomes due to its inadequacy in accurately considering factors such as the area, time period, and age range of the study population^30,32,33^. Many studies have performed statistical analysis to extract their own sets of coefficients for TRISS based on the local context^30^. In the present study, first, we used a conventional coefficient for calculating the probability of survival, named TRISS; second, we utilized another set of coefficients, employed in another interesting study by Domingues et al. and called it aTRISS^9^. Furthermore, we evaluated another scoring system, named the NTRISS, which was developed by Domingues in 2018 and establishes the SBP, NISS, and best motor response, instead of the RTS and ISS, in the conventional TRISS^9^. There were several concerns, given the use of the TRISS with conventional coefficients in children; nevertheless, in a recently published study in 2023 in which 11 models of the TRISS were evaluated for pediatric trauma, the authors concluded that the proposed models are not superior to conventional models^30^.

A study conducted in Turkey examined 1510 patients with an average age of 7.8 years and a mortality rate of 4.2%. That study evaluated the performance of the NISS, ISS, GCS, and RTS in predicting in-hospital mortality, with each scoring system having an impressive AUC of 0.993, 0.992, 0.987, and 0.976, respectively. Additionally, these measures achieved AUCs of 0.936, 0.934, 0.913, and 0.903 for predicting ICU admission. However, it is important to note that the study was conducted in a single center, which may limit the generalizability of the findings. Furthermore, the higher mortality rate observed in this particular center should be taken into consideration when interpreting the results^34^.

In the present study, as shown in Table 5, the predictive performance of the GCS for in-hospital mortality was significantly greater than that of the ISS and NISS. In parallel with our findings, in a single-center study of 588 pediatric trauma patients, the GCS had the best predictive performance compared to the ISS^35^. However, these findings were reversed in our study when considering ICU admission as the outcome. The GCS exhibited poorer predictive performance than did the ISS and the NISS.

In a single-center study comprising 74 pediatric Turkish patients with a mean age of 7 years and nearly 90% of whom the injuries were blunt in nature, the RTS, TRISS, and ISS were found to be independent predictors of ICU admission^10^.

In a study conducted at a single center involving 938 pediatric patients with an average age of 3.1 years and a mortality rate of 0.9% in 2019, ISS demonstrated the highest AUC compared to RTS and GCS (AUC = 0.975, 0.899, and 0.864, respectively)^36^. Interestingly, our study showed contrasting results in terms of predictive performance among these three scoring systems. Specifically, we observed that the GCS provided better mortality prediction than did the RTS or ISS (AUC = 0.955, 0.944, and 0.901, respectively). This discrepancy may be attributed to the higher mortality rate in their study and the fact that it was conducted at a single center. Additionally, the inclusion of only children younger than six years in their research is a crucial factor. This strongly suggested that the age of patients significantly influences the use of scoring systems in a pediatric context. Eventually, after choosing cutoff values of 15 for ISS, 11 for GCS, and 7 for RTS in their study, the crude ORs for mortality were 109.25 for patients with ISS ≥ 15, 136.50 for patients with GCS ≤ 11, and 94.72 for patients with RTS ≤ 7^36^. In our study, the crude ORs for patients with RTS < 7 were 104.40 and 216.21 for GCS score ≤ 8.

In contrast to many other studies that simply utilize predetermined cut-offs from existing literature to calculate the AUC for scoring systems, our current study took a different approach. Initially, we treated all scoring systems as continuous variables and constructed ROC curves for each. Subsequently, we utilized our own findings in conjunction with those from existing literature to determine cut-off values for the scoring systems that effectively categorized patients with both specificity and sensitivity in relation to measured outcomes. Our study successfully demonstrated that all identified cut-off values for the scoring systems were able to significantly differentiate patients based on their outcomes.

An effective scoring system that can accurately assess the severity and type of injury is crucial for prioritizing patient treatment, forecasting patient recovery, assessing trauma care, and distributing therapeutic resources. Selecting an appropriate trauma scoring system is crucial for its effective application based on the context. The application of these trauma scoring systems to guarantee their effectiveness and proper utilization should be comprehended^37^.

Based on the literature, an AUC ≥ 0.80 is considered as being an acceptable diagnostic test, and an AUC ≥ 0.90 is being excellent one^38^. Regarding the in-hospital mortality as the main outcome of this study, there might be some degrees of statistical difference among scoring systems, which can be explained by the large number of patients included in this study. Nonetheless, except for the AIS, all scoring systems had excellent results in predicting mortality (AUC ≥ 0.90). In other words, these scoring systems are clinically excellent scoring systems in the prediction of in-hospital mortality. Given the ICU admission as the second outcome in this study, TRISS and its two variants, as well as ISS and NISS had an acceptable performance in its prediction (AUC ≥ 0.80), although none of them had an excellent performance (AUC ≥ 0.90). Despite of statistical difference among them, these five scoring systems are acceptable systems for ICU admission prediction.

In summary, it is imperative to acknowledge that relying solely on anatomical or physiological factors for predicting pediatric trauma cases may be inadequate. However, adopting a combinatorial approach that incorporates both of these factors simultaneously can enhance the accuracy of outcome prediction. This is precisely why TRISS, along with its two other variants, yielded the most appealing outcomes.

## Study limitations and considerations

The AIS scores utilized in this study were generated manually. Previous researchers have reported discrepancies between AIS scores assigned by machines and those assigned manually^39^. The considerable number of included pediatric trauma patients and the multicenter nature of this study increase the generalizability of the findings.

## Conclusion

In this study, we demonstrated that TRISS and its two derivatives, namely, aTRISS and NTRISS, have the potential to effectively identify patients who are at a greater risk of experiencing adverse outcomes. In addition, we showed that anatomical-based scoring systems have better predictive performance for ICU admission than their physiological counterparts; however, physiological-based scoring systems are better predictors of in-hospital mortality. These findings highlight the importance of utilizing these measures in clinical practice to improve patient care and patient prognosis.

## Supplementary Information

Supplementary Information.

## Data Availability

All essential data have been included in this manuscript. Further data can be accessed via the corresponding author upon reasonable request.

## References

[1] Borse, N. & Sleet, D. A. CDC childhood injury report: Patterns of unintentional injuries among 0-to 19-year olds in the United States, 2000–2006. *Fam. Community Health* **32**, 189 (2009). 19305217 10.1097/01.FCH.0000347986.44810.59

[2] Theodorou, C. M. *et al.* Causes of early mortality in pediatric trauma patients. *J. Trauma Acute Care Surg.* **90**, 574 (2021). 33492107 10.1097/TA.0000000000003045 PMC8008945

[3] Shi, J. *et al.* A new weighted injury severity scoring system: Better predictive power for pediatric trauma mortality. *J. Trauma Acute Care Surg.* **85**, 334 (2018). 29787558 10.1097/TA.0000000000001943 PMC6081250

[4] Mora, M. C. *et al.* Pediatric trauma triage: A Pediatric Trauma Society Research Committee systematic review. *J. Trauma Acute Care Surg.* **89**, 623–630. 10.1097/ta.0000000000002713 (2020). 32301877 10.1097/TA.0000000000002713

[5] Beuran, M. *et al.* Trauma scores: A review of the literature. *Chirurgia* **107**, 291–297 (2012). 22844826

[6] Roy, N. *et al.* Validation of international trauma scoring systems in urban trauma centres in India. *Injury* **47**, 2459–2464 (2016). 27667119 10.1016/j.injury.2016.09.027

[7] Soni, K. D. *et al.* Comparison of ISS, NISS, and RTS score as predictor of mortality in pediatric fall. *Burns Trauma* **5**, 25 (2017). 28795055 10.1186/s41038-017-0087-7 PMC5547492

[8] Jalilvand, H. *et al.* Prognostic value of Prehospital Index (PHI) versus GAP scale measured in hospital emergency room. *Med. Sci.* **36**, 2126 (2021).

[9] Domingues, C. A., Coimbra, R., Poggetti, R. S., Nogueira, L. S. & de Sousa, R. M. C. New Trauma and Injury Severity Score (TRISS) adjustments for survival prediction. *World J. Emerg. Surg.* **13**, 1–6 (2018). 29541155 10.1186/s13017-018-0171-8 PMC5840784

[10] Narcı, A. *et al.* The prognostic importance of trauma scoring systems in pediatric patients. *Pediatr. Surg. Int.* **25**, 25–30 (2009). 19009298 10.1007/s00383-008-2287-5

[11] Sullivan, T. *et al.* Prediction of mortality in pediatric trauma patients: New injury severity score outperforms injury severity score in the severely injured. *J. Trauma Acute Care Surg.* **55**, 1083–1088 (2003). 10.1097/01.TA.0000102175.58306.2A 14676655

[12] Marcin, J. P. & Pollack, M. M. Triage scoring systems, severity of illness measures, and mortality prediction models in pediatric trauma. *Crit. Care Med.* **30**, S457–S467 (2002). 12528788 10.1097/00003246-200211001-00011

[13] States, J. D. The abbreviated and the comprehensive research injury scales. *SAE Transactions* 2625–2634 (1969).

[14] Teasdale, G. & Jennett, B. Assessment of coma and impaired consciousness: A practical scale. *Lancet* **304**, 81–84 (1974). 10.1016/s0140-6736(74)91639-0 4136544

[15] Baker, S. P., O’Neill, B., Haddon, W. Jr. & Long, W. B. The injury severity score: A method for describing patients with multiple injuries and evaluating emergency care. *J. Trauma Acute Care Surg.* **14**, 187–196 (1974). 4814394

[16] Champion, H. R., Sacco, W. J., Carnazzo, A. J., Copes, W. & Fouty, W. J. Trauma score. *Crit. Care Med.* **9**, 672–676 (1981). 7273818 10.1097/00003246-198109000-00015

[17] Koehler, J. J. *et al.* Prehospital Index: A scoring system for field triage of trauma victims. *Ann. Emerg. Med.* **15**, 178–182 (1986). 3946860 10.1016/s0196-0644(86)80016-6

[18] Champion, H. R. *et al.* A revision of the trauma score. *J. Trauma Acute Care Surg.* **29**, 623–629 (1989). 10.1097/00005373-198905000-00017 2657085

[19] Osler, T., Baker, S. P. & Long, W. A modification of the injury severity score that both improves accuracy and simplifies scoring. *J. Trauma Acute Care Surg.* **43**, 922–926 (1997). 10.1097/00005373-199712000-00009 9420106

[20] Zagory, J. A. *et al.* Evaluation of highest level pediatric trauma activation criteria. *Pediatr. Emerg. Care* **34**, 787–790 (2018). 28538607 10.1097/PEC.0000000000001178

[21] Drendel, A. L., Gray, M. P. & Lerner, E. B. A systematic review of hospital trauma team activation criteria for children. *Pediatr. Emerg. Care* **35**, 8 (2019). 30608908 10.1097/PEC.0000000000001256 PMC6913171

[22] Safety, C. o. M. A. o. A. Rating the severity of tissue damage. I. The abbreviated scale. *JAMA* **215**, 277–280 (1971). 10.1001/jama.1971.03180150059012 5107365

[23] Brown, J. B. *et al.* The value of the injury severity score in pediatric trauma: Time for a new definition of severe injury?. *J. Trauma Acute Care Surg.* **82**, 995 (2017). 28328674 10.1097/TA.0000000000001440 PMC5464600

[24] Tohira, H., Jacobs, I., Mountain, D., Gibson, N. & Yeo, A. Systematic review of predictive performance of injury severity scoring tools. *Scand. J. Trauma Resusc. Emerg. Med.* **20**, 1–12 (2012). 22964071 10.1186/1757-7241-20-63 PMC3511252

[25] Grisoni, E. *et al.* The New Injury Severity Score and the evaluation of pediatric trauma. *J. Trauma Acute Care Surg.* **50**, 1106–1110 (2001). 10.1097/00005373-200106000-00021 11428379

[26] Manoochehry, S., Vafabin, M., Bitaraf, S. & Amiri, A. A comparison between the ability of revised trauma score and Kampala trauma score in predicting mortality; A meta-analysis. *Arch. Acad. Emerg. Med.* **7**, e6 (2019). 30847441 PMC6377219

[27] Muisyo, T. *et al.* Mortality prediction in pediatric trauma. *J. Pediatr. Surg.* **54**, 1613–1616 (2019). 30270118 10.1016/j.jpedsurg.2018.08.045

[28] Cicero, M. X. & Cross, K. P. Predictive value of initial Glasgow coma scale score in pediatric trauma patients. *Pediatr. Emerg. Care* **29**, 43–48 (2013). 23283262 10.1097/PEC.0b013e31827b52bf

[29] Nakhjavan-Shahraki, B. *et al.* Performance of physiology scoring systems in prediction of in-hospital mortality of traumatic children: A prospective observational study. *J. Clin. Orthop. Trauma* **8**, S43–S48. 10.1016/j.jcot.2017.08.001 (2017). 29158647 10.1016/j.jcot.2017.08.001 PMC5681232

[30] Toida, C. *et al.* Validation of the conventional trauma and injury severity score and a newly developed survival predictive model in pediatric patients with blunt trauma: A nationwide observation study. *Children* **10**, 1542 (2023). 37761503 10.3390/children10091542 PMC10529461

[31] Champion, H. R. *et al.* The major trauma outcome study: Establishing national norms for trauma care. *J. Trauma Acute Care Surg.* **30**, 1356–1365 (1990). 2231804

[32] Kimura, A., Chadbunchachai, W. & Nakahara, S. Modification of the Trauma and Injury Severity Score (TRISS) method provides better survival prediction in Asian blunt trauma victims. *World J. Surg.* **36**, 813–818 (2012). 22354490 10.1007/s00268-012-1498-z

[33] Schall, L. C., Potoka, D. A. & Ford, H. R. A new method for estimating probability of survival in pediatric patients using revised TRISS methodology based on age-adjusted weights. *J. Trauma Acute Care Surg.* **52**, 235–241 (2002). 10.1097/00005373-200202000-00006 11834981

[34] Sultanoğlu, H., Özkan, S., Sultanoğlu, T. E. & Kavak, N. Comparison of trauma scoring systems in pediatric trauma patients. *Euras. J. Emerg. Med.* **18**, 1–8 (2019).

[35] Yousefzadeh-Chabok, S. *et al.* Comparing Pediatric Trauma, Glasgow Coma Scale and Injury Severity scores for mortality prediction in traumatic children. *Turk. J. Trauma Emerg. Surg.* **22**, 328–332 (2016). 10.5505/tjtes.2015.83930 27598603

[36] Huang, Y.-T., Huang, Y.-H., Hsieh, C.-H., Li, C.-J. & Chiu, I.-M. Comparison of Injury Severity Score, Glasgow Coma Scale, and Revised Trauma Score in predicting the mortality and prolonged ICU stay of traumatic young children: A cross-sectional retrospective study. *Emerg. Med. Int.* **2019**, 5453624 (2019). 31885926 10.1155/2019/5453624 PMC6914995

[37] Kim, Y. J. Injury severity scoring systems: A review of application to practice. *Nurs. Crit. Care* **17**, 138–150 (2012). 22497918 10.1111/j.1478-5153.2012.00498.x

[38] Nahm, F. S. Receiver operating characteristic curve: Overview and practical use for clinicians. *Korean J. Anesthesiol.* **75**, 25–36. 10.4097/kja.21209 (2022). 35124947 10.4097/kja.21209 PMC8831439

[39] Di Bartolomeo, S., Tillati, S., Valent, F., Zanier, L. & Barbone, F. ISS mapped from ICD-9-CM by a novel freeware versus traditional coding: A comparative study. *Scand. J. Trauma Resusc. Emerg. Med.* **18**, 1–7 (2010). 20356359 10.1186/1757-7241-18-17 PMC2852374

