# Supplementary material for: Comparison of nine trauma scoring systems in prediction of inhospital outcomes of pediatric trauma patients: a multicenter study
Source: Sci Rep. 2024 Apr 1;14:7646. doi: 10.1038/s41598-024-58373-4 (PMC10985103; doi:10.1038/s41598-024-58373-4)
Supplement: Supplementary file 1 — Supplementary Information. [file 41598_2024_58373_MOESM1_ESM.docx]

The Revised Trauma Score (RTS), as a comprehensive physiological scoring system, takes into account three vital variables: Glasgow Coma Scale (GCS), Systolic Blood Pressure (SBP), and Respiratory Rate (RR). Each variable is assigned a coded value ranging from 0 to 4. The final RTS value, determined through the following formula, ranges from 0 to 7.8408 (1):

*RTS = 0.9368 GCS + 0.7326 SBP + 0.2908 RR*

This study compared various models of survival probability, all of which utilized physiological parameters obtained upon patient admission to the hospital. The survival probabilities were calculated using a specific equation *P_s_ =*$(\frac{1}{1+e^{-b}})$*;* where *P_s_* is the probability of survival and *e* =2.718282 (base of the Neperian logarithm). The values of b varied across three TRISS-derived models, as outlined below:

TRISS; *b=b_0_+b_1_ (RTS) +b_2_ (ISS) +b_3_ (age index) ^*^*

The RTS index's total value ranges from 0 to 7.84. For the age index, a value of 0 is assigned if the age is less than 55 years, while a value of 1 is assigned if the age is 55 years or older. As in this study all cases were $\leq$18 years, and the age index was zero. *b* coefficients are presented in detail, based on penetrating or blunt injuries in ***Supplementary Table 1***.

aTRISS; *b=b_0_+b_1_ (RTS) +b_2_ (ISS) +b_3_ (age index) ^*^*

The RTS index's total value ranges from 0 to 7.84. For the age index, a value of 0 is assigned if the age is less than 55 years, while a value of 1 is assigned if the age is 55 years or older. As in this study all cases were $\leq$18 years, and the age index was zero. *b* coefficients are presented in detail, based on penetrating or blunt injuries in ***Supplementary Table 1,*** and are retrieved from another study by Domingues *et. al* (2).

NTRISS; *b=b_0_+b_1_ (BMR) + b_2_ (SBP) + b_3_ (NISS) + b_4_ (age index) **

The best motor response (BMR)’s assigned value is based on the GCS motor response, ranging from 1 to 6. Similarly, SBP is the value attributed to this parameter in the RTS, ranging from 0 to 4. Moreover, the Age variable is assigned a value of 0 if the individual is younger than 55 years, and 1 if the individual is 55 years or older. As in this study all cases were $\leq$18 years, and the age index was zero. *b* coefficients are presented in detail, based on penetrating or blunt injuries in ***Supplementary Table 1,*** and are retrieved from another study by Domingues *et. al* (2).

The prehospital index (PHI) was determined by summing the values assigned to various vital signs and injury characteristics (3). These included SBP, pulse rate, respiratory rate, level of consciousness, and the type of injury (penetrating or blunt). For SBP, a value of 0 was assigned if it exceeded 100, 1 if it ranged from 86 to 100, 2 if it ranged from 75 to 85, and 5 if it was between 0 and 74. Pulse rates above 120 received a score of 3, while rates below 50 received a score of 5. Any pulse rate within this range received a score of zero. Patients with normal respiration were assigned a score of zero, those with labored or shallow respiration received a score of 3, and individuals with less than 10 breaths per minute or requiring intubation received a score of 5. The level of consciousness was also taken into account. Confused or combative speech received a score of 3, while incoherent speech without intelligible words received a score of 5. Lastly, the type of injury was considered. Penetrating injuries were assigned a score of 4, while blunt injuries received no score.

The ISS and NISS were calculated retrospectively, using internationally agreed-upon definitions for Abbreviated Injury Score (AIS) coding. Each patient's injuries were coded based on the AIS, and the highest values from the three regions were chosen. These highest values were then squared and summed to determine the ISS. Likewise, the top three scores, irrespective of body regions, were squared and summed to derive a composite score referred to as NISS.

| **Supplementary table 1. Coefficients of TRISS, aTRISS, and NTRISS-like** | | |
| --- | --- | --- |
| TRISS; *P_s_ =*$(\frac{1}{1+e^{-b}})$*;* where *b=b_0_+b_1_ (RTS) +b_2_ (ISS) +b_3_ (age index)^*^* | | |
| Coefficients | Blunt | Penetrating |
| B_0_ | -0.4499 | -2.5355 |
| B_1_ | 0.8085 | 0.9934 |
| B_2_ | -0.0835 | -0.0651 |
| B_3_ | -1.7430 | -1.1360 |
| aTRISS; *P_s_ =*$(\frac{1}{1+e^{-b}})$*;* where *b=b_0_+b_1_ (RTS) +b_2_ (ISS) +b_3_ (age index)^*^* | | |
| Coefficients | Blunt | Penetrating |
| B_0_ | -1.6479 | -1.2980 |
| B_1_ | 0.9053 | 0.8953 |
| B_2_ | -0.0784 | -0.0952 |
| B_3_ | -1.3801 | -1.2754 |
| NTRISS; *P_s_ =*$(\frac{1}{1+e^{-b}})$*;* where *b=b_0_+b_1_ (BMR) + b_2_ (SBP) + b_3_ (NISS) + b_4_ (age index)** | | |
| Coefficients | Blunt | Penetrating |
| B_0_ | -1.6760 | -1.5863 |
| B_1_ | 0.6194 | 0.5888 |
| B_2_ | 0.8953 | 0.9695 |
| B_3_ | -0.07289 | -0.0665 |
| B_4_ | -1.3308 | -1.0058 |
| TRISS: Trauma and Injury severity score; aTRISS: adjusted Trauma and Injury severity score; NTRISS: New Trauma and Injury severity score; GCS: Glasgow coma scale; ISS: injury severity score; NISS: new injury severity score; BMR: best motor response; RTS: revised trauma score; SBP: systolic blood pressure  * Age index=0 for all cases, as age is <55 years. | | |

References:

1. Champion HR, Sacco WJ, Copes WS, Gann DS, Gennarelli TA, Flanagan ME. A revision of the Trauma Score. Journal of Trauma and Acute Care Surgery. 1989;29(5):623-9.

2. Domingues CdA, Coimbra R, Poggetti RS, Nogueira LdS, de Sousa RMC. New Trauma and Injury Severity Score (TRISS) adjustments for survival prediction. World journal of emergency surgery. 2018;13:1-6.

3. Koehler JJ, Baer LJ, Malafa SA, Meindertsma M, Navitskas NR, Huizenga JE. Prehospital Index: a scoring system for field triage of trauma victims. Annals of emergency medicine. 1986;15(2):178-82.
